# The Relationship Between Exercise Intention and Exercise Behavior of Junior School Students: An Analysis of Chain Mediating Effect

**DOI:** 10.3389/fpsyg.2022.935264

**Published:** 2022-08-08

**Authors:** Yue Chen, Shu-Jun Yao, Qi-Shuai Ma, Wei Shao, Chao Liu, Ke-Lei Guo

**Affiliations:** ^1^School of Physical Education, Huaibei Normal University, Huaibei, China; ^2^School of Physical Education and Health, Zhaoqing University, Zhaoqing, China

**Keywords:** exercise intention, implementation intentions, self-identity, exercise behavior, junior school students

## Abstract

**Objective:**

This study explores the relationship between exercise intention and exercise behavior and constructs a chain mediating model through the mediating effect of implementation intention and self-identity.

**Method:**

Through the stratified cluster sampling method, 1,573 junior school students (with an average age of 13.71 ± 0.891 years) were evaluated by the exercise intention scale, the implementation intention scale, the self-identity scale, and the physical exercise grade scale. For data analysis, the common method deviation test, Pearson correlation analysis, and Model 6 in the SPSS macro program compiled by Hayes for the chain mediating test were conducted.

**Results:**

(1) There is a marked correlation between positive exercise intention and exercise behavior (*r* = 0.345, *p* < 0.01), and exercise intention has a significant effect on the direct path of exercise behavior (β = 0.162, *t* = 12.355, *p* < 0.01). (2) Exercise intention can positively predict implementation intention (β = 0.219, *t* = 10.006, *p* < 0.01) and self-identity (β = 0.160, *t* = 16.159, *p* < 0.01); implementation intention can significantly and positively predict exercise behavior (β = 0.230, *t* = 12.742, *p* < 0.01),and self-identity can significantly and positively predict exercise behavior (β = 0.273, *t* = 7.911, *p* < 0.01). (3) Implementation intention and self-identity play a significant mediating role between exercise intention and exercise behavior. The mediating effect consists of three indirect effects: exercise intention → implementation intention → exercise behavior (the mediating effect value is 0.050), exercise intention → self-identity → exercise behavior (the mediating effect value is 0.044), and exercise intention →implementation intention → self-identity → exercise behavior (the mediating effect value is 0.017).

**Conclusion:**

(1) Exercise intention can significantly and positively predict exercise behavior. (2) Implementation intention and self-identity exert a significant mediating effect between exercise intention and exercise behavior, including the separate mediating effect of implementation intention and self-identity, as well as the chain mediating effect of implementation intention and self-identity.

## Introduction

In recent years, the physical health of teenagers has attracted extensive attention from all walks of life. Since 1985, China has carried out the national adolescent physical health survey. The results show that the physical fitness of Chinese adolescents has continued to decline in the past 20 years, and the prevalence of obesity among students has continued to increase (Wu and Yuan, 2019). According to the results of the 8th National Survey on Students’ Physical Condition and Health released by the Ministry of Education (2021), the excellent and good rate of students’ physique and health was only 23.8%. Therefore, the healthy development of teenagers’ physique is still not optimistic. Existing studies have shown that a sedentary lifestyle and lack of physical activity have become recognized social problems in the world today and are also risk factors leading to the rise of obesity and myopia. Especially for junior middle school students facing the “high school entrance examination,” their academic burden accounts for most of their physical exercise time, and they lack healthy exercise activities (Zhang and Mao, 2016).

A large number of studies have confirmed that the reduction of exercise behavior is the main reason for the decline of teenagers’ physique, the rising overweight and obesity rates (Liu et al., 2022), and the increasing myopia (Yang et al., 2017; Guo, 2019). Exercise behavior refers to a specific way of behavior to achieve the purpose of physical exercise (Yan et al., 2020). The benefits of exercise behavior and physical health have been confirmed in previous studies (Harada, 2022). For example, regular physical exercise can reduce the incidence rate of chronic diseases and control the incidence of overweight and the risk of mental diseases (Wang and Zheng, 2020). Junior middle school students are in puberty, which is a golden period for individuals to form good skills and exercise habits. Improving exercise behavior can help junior middle school students adhere to physical exercise for a long time, so as to develop physical exercise habits (Lee and Lee, 2020). Therefore, guiding junior middle school students to improve their participation in physical exercise and develop exercise habits has become an important part of improving junior middle school students’ physical health. At present, the existing reasons affecting junior middle school students’ exercise behavior are diverse, including macro-social factors, meso school factors (Leo et al., 2022), family factors, and micro individual factors. However, irrespective of the level of factors, their influence path is from the macrolevel to the microlevel, or exists as an intermediary variable or a third variable. Therefore, in order to reveal the internal mechanism of junior school students’ exercise behavior, it is necessary to further clarify the specific path of intention to behavior.

The theory of planned behavior (TPB), one of the most effective social cognitive theories (Khan et al., 2022a), holds that intention is the direct factor determining behavior (Zhang and Mao, 2016; Bosnjak et al., 2020). Intention refers to individuals’ subjective evaluation of the possibility that they want to engage in a certain behavior in the future, as well as the efforts that they are willing to make in the process of implementing the plan (Fang, 2012), which plays a crucial role in enhancing exercise activities (Cao and Jiang, 2013). In recent years, many scholars have studied the relationship between intention and behavior directly or indirectly from various angles (Ullah et al., 2021; Khan et al., 2022b). In the field of management, a large number of researchers have conducted much research on how to promote the work behavior of enterprise personnel from the perspective of TPB (Fatima et al., 2017; Ali et al., 2018). Research shows that in the workplace, employees’ work willingness can significantly predict their work behavior (Khan et al., 2022c; Zada et al., 2022a), and work willingness is affected by the work environment (Saeed et al., 2022a; Zada et al., 2022b) and their own cognition (Khan et al., 2021; Saeed et al., 2022b). At the same time, the theory has also been widely applied in the field of exercise behavior (Conner et al., 2010). A large number of studies have shown that exercise intention can affect exercise behavior (Fang and Sun, 2010; Xu et al., 2018; Lee et al., 2019), and intervention behavior intention can increase junior high school students’ exercise activities, so as to develop a regular exercise lifestyle (Hagger, 2018). Turning intention into practical action and bridging the gap between intention and behavior have become a heated issue in the research and application of TPB in the field of exercise. Therefore, with the aim to continue to improve the predictive explanatory power of behavior, this study explores the process of the transformation from intention to behavior so as to identify other key influencing factors of intention and behavior. In summary, exercise intention can improve students’ exercise behavior and promote the development of physical and mental health. Thus, we propose the following hypothesis.

**H1:** There is a positive relationship between exercise intention and exercise behavior.

### Mediating Effect of Implementation Intention

Implementation intention refers to a specific action plan that specifies where, when, and how individuals will take action to achieve their goals. It is designed as a will strategy to promote the transformation of intention into action, which helps individuals overcome the “intention–behavior” gap (Alexander and Andreas, 2021). Implementation intention connects the specific situation (where and when) of the action with the preselected goal-oriented action in the form of “if–then,” so as to effectively promote the realization of the goal. According to the stimulus–response (S-R) theory, implementation intention is an effective means to change behavior by forming a S-R connection (Adriaanse et al., 2009). Scholars believe that the implementation of the act of will requires a series of action control. If variables enabling individuals to subjectively feel a sense of control and ability over behavior are added between intention and behavior (Feng and Mao, 2014), the gap between intention and behavior can be bridged (Schuz et al., 2006). One of the mediating mechanisms of this study is the mediating effect of implementation intention. In the “if–then” plan, implementation intention connects situational clues and goal-oriented response. A sense of control over behavior enables individuals to be clearly aware of when, where, and what actions to take to achieve their goals, thus assisting the individuals to match exercise plans with situational clues, so that the explanatory power of exercise behavior can be lifted (Wang and Wang, 2021). The formation of implementation intention makes a strong correlation between the expected situation and the target behavior and thus becomes a mediating variable to establish the relationship between intention and behavior (Pfeffer and Strobach, 2017). Previous studies have shown that in the process of physical activity, behavior changes in teenagers who merely changed their intentions were far from satisfactory (Wang et al., 2014), while there was a fairly high probability for teenagers who engaged in implementation intention to overcome immediate difficulties and to automate their behavior intention after deliberation, so as to transform physical activity intention into actual participation behavior (Wang and Zheng, 2020). This shows that increasing intention does not necessarily mean increasing behavior, and this process will also be disturbed by the third variable. Therefore, we speculate that implementation intention may play a crucial role in the process of junior middle school students’ exercise intention transforming into exercise behavior. That is, when junior middle school students have exercise intention, they need a certain level of executive power to truly transform exercise intention into exercise behavior. Thus, we propose the following hypothesis.

**H2:** Implementation intention has a mediating effect between exercise intention and exercise behavior of junior school students.

### Mediating Effect of Self-Identity

In the existing research on exercise behavior, the determination of mediating variables is mainly based on the theoretical guidance in the field of this discipline or deduced from the relevant research results, but there is still a lack of multidisciplinary perspective on the determination of mediating variables. Another mediating mechanism that this study focuses on is the mediating effect of self-identity, which refers to the reflective understanding of the self formed by individuals based on their experiences. There is internal consistency and continuity before and after individual development (Bixter et al., 2020). Self-identity is not a stable trait. It will be tested and reflected with personal experience. It is the result of the continuous development of individuals (Fatt et al., 2021). The essence of self-identity is to solve the problem of “who am I,” and it is the internal motivation of individual cognitive development. Individual cognition plays an important role in the TPB. According to the TPB, behavior is the result of individual deliberate planning. The higher the individual’s cognitive level, the stronger the perceptual behavior control of behavior (Rezaei et al., 2021). Therefore, we use TPB as a reference, and self-identity as an intermediary variable. Research shows that self-identity can effectively predict the intention to participate in sports in the next 2 months (Soekmawati et al., 2022). Another study has found that self-identity plays a partial mediating role in the relationship between exercise intention and exercise behavior of junior school students. Self-identity can directly predict behavior or indirectly affect behavior through intention, and is considered a significant predictor (Jackson et al., 2003). In other words, a higher level of self-identity means that individuals have stronger internal motivation. This internal motivation of eagerness to improve their own value will promote the transformation of junior high school students’ exercise intention to exercise behavior. Thus, we propose the following hypothesis.

**H3:** Self-identity has a mediating effect between exercise intention and exercise behavior of junior school students.

### Chain Mediating Effect of Implementation Intention and Self-Identity

The personality development theory holds that if individuals get positive results when integrating past experience, higher self-identity will be generated (Chen and Yu, 2020). Some studies believe that implementation intention has a sense of control over behavior (Manuel et al., 2021), which may significantly lift individual self-identity. A higher level of implementation intention helps teenagers to achieve their goals, and in addition, the praise obtained after completing the goals will further lead to a higher sense of self-identity (Feng et al., 2020). Therefore, there may be a significant positive correlation between implementation intention and self-identity. Implementation intention plays a mediating role between exercise intention and exercise behavior, while self-identity may further accelerate this mediating process. In the process of transforming exercise intention into exercise behavior, it is necessary to control behavior through implementation intention, which may increase exercise behavior through self-identity. To sum up, exercise intention can predict teenagers’ implementation intention, which exerts an impact on self-identity, and self-identity may affect the formation of exercise behavior. In view of this discussion, we propose the following hypothesis.

**H4:** Implementation intention and self-identity play a chain mediating role between exercise intention and exercise behavior.

**FIGURE 1:**
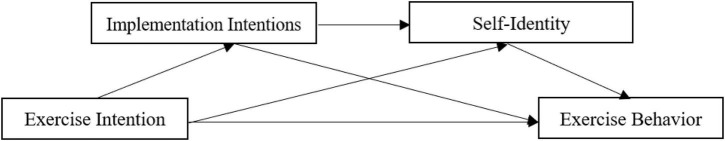
Conceptual framework.

To sum up, in order to investigate the internal mechanism between exercise intention and exercise behavior, this study intends to build a chain mediating model (as shown in Figure 1) and verify the following aspects: (1) Exercise intention significantly and positively predicts junior school students’ exercise behavior; (2) implementation intention plays an independent mediating role between exercise intention and exercise behavior of junior school students; (3) self-identity plays an independent mediating role between exercise intention and exercise behavior of junior school students; and (4) implementation intention and self-identity play a chain mediating role between exercise intention and exercise behavior of junior school students.

## Materials and Methods

### Participants and Procedure

According to the administrative division of Anhui Province in China and the different levels of regional economic development, one junior school was selected, respectively, from the cities and rural areas in the south, middle, and north of Anhui Province. In 2021, with stratified sampling, two classes were randomly selected from each grade of urban and rural schools, with a total of 36 classes, and 1,650 junior school students filled in the questionnaire. As for the collection, 77 invalid questionnaires with regular answers and a lack of data were excluded, with a recovery rate of 95.33%. Totally, 1,573 valid questionnaires were obtained, among which there were 746 boys and 827 girls, 704 students in grade 1, 407 students in grade 2, and 462 students in grade 3. The average age of the subjects was 13.71 ± 0.891 years. The completion time of each questionnaire was about 5–8 min.

The test was conducted with the consent of the head teacher, parents, and subjects themselves. The experimenters are college students majoring in sports psychology. The collective test was adopted, emphasizing the principles of anonymous filling, data confidentiality, and voluntary filling. Being in accordance with the Declaration of Helsinki, the research design has passed the ethical review procedure of the human research ethics committee of Huaibei Normal University. In this process, all invited participants were voluntary; thus, confidentiality has been guaranteed, and written informed consent of all participants’ parents or guardians has been obtained.

### Measures

A description of the measures used in the data collection for the current analyses is given in the following text. All questionnaire items are in Appendix.

### Exercise Intention

The Chinese version of the *Exercise Intention Scale* compiled by Ajzen (2006) and translated and revised by Hu (2008) has been adopted to measure the exercise intention of junior school students. The study confirms that the scale is suitable for measuring the exercise intention of Chinese junior school students (Fang, 2012). The dimension of the scale is composed of three items, with an aim to assess the willingness and possibility of teenagers to participate in exercise (e.g., “In the next two weeks, I plan to take physical exercise for more than 20 minutes at least three times a week”). The scale adopts a 7-point Likert score, ranging from “1 = strongly disagree” to “7 = strongly agree.” The higher the score, the stronger the intention to participate in exercise. In this study, the three items of the *Exercise Intention Scale* converged well to one factor, with a KMO value of 0.91 and a chi-square value of the Bartlett ball test of 186,253.88 (*p* < 0.01), explaining 74.32% of the total variance (Table 1). The fitting index of confirmatory factor analysis is χ^2^/*df* = 2.792, *CFI* = *0.932*, *NFI* = *0.911*, *GFI* = *0.917*, *NNFI* = *0.925*, and *RMESA* = *0.093*. The goodness-of-fit is significantly better, indicating that the scale has good structural validity (Table 2). Cronbach’s α is 0.79, indicating that the internal consistency of the scale is good.

**TABLE 1:** Results of exploratory factor analysis and internal consistency test.

Factor naming	KMO	Bartlett Chi-square value (*P*-value)	Cumulative variance interpretation rate	Cronbach’ coefficient
Exercise Intention	0.91	186253.88 (*p* < 0.01)	74.32%	0.79
Implementation Intention	0.94	8860.44 (*p* < 0.01)	73.75%	0.90
Self-identity	0.93	3453.77 (*p* < 0.01)	70.92%	0.73
Exercise Behavior	0.90	274060.11 (*p* < 0.01)	64.84%	0.72

**TABLE 2:** Results of confirmatory factor analysis.

Factor naming	χ^2^/*df*	*CFI*	*NFI*	*GFI*	*NNFI*	*RMESA*
Exercise Intention	2.792	0.932	0.911	0.917	0.925	0.093
Implementation Intention	2.431	0.961	0.933	0.964	0.917	0.083
Self-identity	1.885	0.991	0.984	0.990	0.906	0.061
Exercise Behavior	2.332	0.984	0.931	0.906	0.981	0.037

### Implementation Intention

The Chinese version of the *Implementation Intention Scale* compiled by Brickell et al. (2006) and revised by Wang and Zheng (2020) has been used to measure the implementation intention of junior school students. The dimension of the scale is composed of six items, aiming to understand the plan and implementation of participants in terms of the time, place, and method of participating in physical activities. The more sufficient the plan and implementation, the higher the implementation intention (e.g., “I have planned to take part in physical exercise next week”). The scale adopts the 7-point Likert score, ranging from “1 = strongly disagree” to “7 = strongly agree.” The higher the score, the stronger the execution intention. A previous study has proved that the scale has been well applied among Chinese junior school students (Wang and Zheng, 2020). In this study, the six items of the *Implementation Intention Scale* converged well to one factor, with a KMO value of 0.94 and a chi-square value of the Bartlett ball test of 8,860.44 (*p* < 0.01), accounting for 73.75% of the total variance (Table 1). The fitting index of confirmatory factor analysis is χ^2^/*df* = 2.431, *CFI* = *0.961*, *NFI* = *0.933*, *GFI* = *0.964*, *NNFI* = *0.917*, and *RMESA* = *0.083*. The goodness-of-fit is significantly better, indicating that the scale has good structural validity (Table 2). Cronbach’s α is 0.90, indicating that the internal consistency of the scale is good.

### Self-Identity

The self-identity of junior school students has been measured by adopting the Chinese version of the *Self-identity on Exercise Scale*, which is compiled by Dong (2013) with reference to the research on the exercise self-identity scale by Theodorakis et al. (1995) and Jackson et al. (2003). Self-identity reflects the views and feelings of physical exercise (e.g., “If I give up exercise, I will feel very frustrated”). The scale adopts a 5-point Likert score, ranging from “1 = highly inconsistent” to “5 = highly consistent.” The higher the score, the stronger the self-identity. A previous study has proved that the scale has good applicability among Chinese junior school students (Wang and Wang, 2008). In this study, the 12 items of the *Self-identity on Exercise Scale* converged well to one factor, with a KMO value of 0.93 and a Chi-square value of the Bartlett ball test of 3,453.77 (*p* < 0.01), explaining 70.92% of the total variance (Table 1). The fitting index of confirmatory factor analysis is χ^2^/*df* = 1.885, *CFI* = *0.991*, *NFI* = *0.984*, *GFI* = *0.990*, *NNFI* = *0.906*, and *RMESA* = *0.061*. The goodness-of-fit was significantly better, indicating that the scale had good structural validity (Table 2). Cronbach’s α is 0.73, indicating that the internal consistency of the scale is good.

### Exercise Behavior

The *Physical Activity Rating Scale* (PARS-3) compiled by Liang (1994) has been adopted. The scale includes three items to investigate the amount of physical exercise from three aspects: physical exercise intensity, exercise time, and exercise frequency. The higher the score, the higher the amount of physical exercise. Equaling to or less than 19 points indicates a small amount of exercise, 20 ∼ 42 points indicate a medium amount of exercise, and equaling to or greater than 43 points indicates a large amount of exercise. According to previous experience, this study has divided the amount of small physical exercise into two parts: no physical exercise and a small amount of exercise. No physical exercise is equal to or less than four points, while a small amount of exercise is 5 ∼ 19 points. Therefore, the physical exercise in this study is divided into four levels, from “1 = no physical exercise” to “4 = large amount of physical exercise.” A previous study has proved that the scale has good applicability among Chinese junior school students (Wang et al., 2022). In this study, the three items of the *Physical Activity Rating Scale* converged well to one factor, with a KMO value of 0.90 and a chi-square value of the Bartlett ball test of 274060.11 (*p* < 0.01), explaining 64.84% of the total variance (Table 1). The fitting index of confirmatory factor analysis is χ^2^/*df* = 2.332, *CFI* = *0.984*, *NFI* = *0.931*, *GFI* = *0.906*, *NNFI* = *0.981*, and *RMESA* = *0.037*. The goodness-of-fit is significantly better, indicating that the scale has good structural validity (Table 2). Cronbach’s α is 0.72, indicating that the internal consistency of the scale is good.

### Data Analysis

This study has adopted IBM SPSS23.0 and AMOS26.0 statistical software for all data analyses. After the questionnaires were collected, all the data have been processed as follows: (1) Exploratory factor analysis was performed on all scales by SPSS23.0; (2) confirmatory factor analysis was performed on all scales by AMOS26.0; (3) internal consistency was tested for all scales by SPSS23.0; (4) the Harman single-factor method has been adopted for the common method deviation test; (5) Pearson correlation analysis has been applied to calculate the relationship among exercise intention, implementation intention, self-identity, and exercise behavior, and continuous variables of normal distribution are expressed as mean (M) ± standard deviation (SD); (6) the SPSS macro program compiled by Hayes in SPSS23.0 has been used to verify the mediating role of implementation intention and self-identity, respectively, in the relationship between exercise intention and exercise behavior, as well as the chain mediating role of implementation intention and self-identity in the relationship between exercise intention and exercise behavior; and (7) Model 6 in the SPSS macro program compiled by Hayes has been adopted for the chain mediating test. In this study, the significance level is set as *p* < 0.05.

## Results and Analysis

### Common Method Bias Test

Common method bias refers to artifactual covariation between a predictor and a valid scale variable because of the same data source or rater, the same measurement environment, the context of the item, and the characteristics of the item itself. Such artifactual covariation, which can severely confound research results and potentially mislead conclusions, is a systematic error. As the data in this study were all collected through a scale, there may be problems of common method bias. Therefore, this study has adopted the Harman single-factor method to test the common method bias. The test results show that there are six factors with characteristic roots greater than 1 in principal component analysis, and the variation explained by the first factor is 30.69%, which is lower than the critical value of 40%, indicating that there is no serious common method bias in this study.

### Descriptive Statistical and Correlation Analysis of Variables

Table 3 presents the mean score, standard deviation, and correlation coefficient of variables in detail. It can be seen from the data in Table 1 that exercise intention (*r* = 0.345, *p* < 0.01), implementation intention (*r* = 0.562, *p* < 0.01), and self-identity (*r* = 0.539, *p* < 0.01) have a significant and positive correlation with exercise behavior. Exercise intention (*r* = 0.504, *p* < 0.01) and implementation intention (*r* = 0.606, *p* < 0.01) have a significant and positive correlation with self-identity. There is a significant and positive correlation between exercise intention (*r* = 0.286, *p* < 0.01) and implementation intention. The results of correlation analysis have provided preliminary support for the subsequent hypothesis test.

**TABLE 3:** Mean, standard deviation, and correlation coefficient of variables.

	M	SD	1	2	3	4
1. Exercise intention	4.88	2.07	1			
2. Implementation intention	2.92	1.59	0.286**	1		
3. Exercise behavior	23.76	7.98	0.345**	0.562**	1	
4. Self-identity	3.21	0.92	0.504**	0.606**	0.539**	1

*At the level of 0.05, the correlation is significant. **At the level of 0.01, the correlation is significant.

The chain mediating effect model has been verified based on the suggestions of Wen and Ye (2014) on the test of the mediating effect, and the test results are shown in Table 4. According to Table 4, with gender and grade in control, exercise intention can significantly and positively predict junior school students’ exercise behavior, with a total effect of 0.162 (*p* < 0.01) and a direct effect of 0.051 (*p* < 0.01). Therefore, Hypothesis 1 is tenable. When incorporating implementation intention and self-identity into the regression equation, exercise intention can significantly and positively predict implementation intention (β = 0.219, *p* < 0.01) and self-identity (β = 0.160, *p* < 0.01). Implementation intention can significantly and positively predict self-identity (β = 0.292, *p* < 0.01) and exercise behavior (β = 0.230, *p* < 0.01). Self-identity can significantly and positively predict exercise behavior (β = 0.273, *p* < 0.01). Based on these results, exercise intention can still significantly and positively predict exercise behavior (β = 0.051, *p* < 0.01), which leads to the conclusion that implementation intention and self-identity, respectively, play a partial mediating role between exercise intention and exercise behavior. Hypothesis 2 and Hypothesis 3 are supported by data.

**TABLE 4:** Analysis of regression relationship of variables.

Effect	Item	Effect	SE	t	p	LLCI	ULCI
Direct effects	Exercise Intention ⇒ Exercise Behavior	0.051	0.013	3.994	< 0.01	0.026	0.076
Indirect effect Process	Exercise Intention ⇒ Implementation Intention	0.219	0.022	10.006	< 0.01	0.176	0.262
	Exercise Intention ⇒ Self-identity	0.160	0.010	16.159	< 0.01	0.141	0.180
	Implementation Intention ⇒ Self-identity	0.292	0.013	22.560	< 0.01	0.267	0.318
	Implementation Intention ⇒ Exercise Behavior	0.230	0.018	12.742	< 0.01	0.195	0.265
	Self-identity ⇒ Exercise Behavior	0.273	0.035	7.911	< 0.01	0.205	0.340
Total effect	Exercise intention ⇒Exercise Behavior	0.162	0.013	12.335	< 0.01	0.137	0.188

*LLCI refers to the lower limit of 95% interval of bootstrap sampling, and ULCI refers to the upper limit of 95% interval of bootstrap sampling.*

**FIGURE 2:**
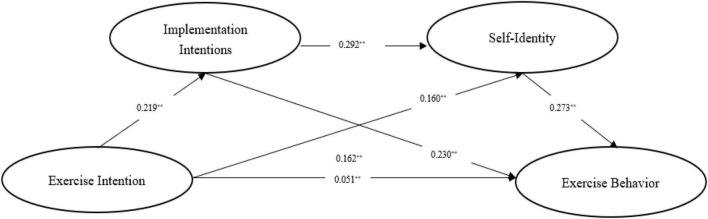
Chain mediating model of implementation intention and self-identity in exercise intention and exercise behavior. **p < 0.01.

The bootstrap mediating test method (Cole et al., 2008) has been adopted to further test the mediating effect, and the process plug-in has been applied to construct the structural equation model. In the process plug-in, Model 6 was selected to test the chain mediating effect of implementation intention and self-identity. The test results are shown in Table 5. The confidence interval of the mediating effect test of implementation intention in exercise intention and exercise behavior is (0.038, 0.067), excluding 0, so that the mediating effect is verified, proving Hypothesis 2 tenable, in which the mediating effect accounts for 30.86% of the total effect. The confidence interval of the mediating effect test of self-identity in exercise intention and exercise behavior is (0.028, 0.060), excluding 0. The mediating effect accounts for 27.16% of the total effect, which is lower than the mediating effect of implementation intention, and proves that Hypothesis 3 is tenable. The confidence interval of the chain mediating effect path of implementation intention and self-identity excludes number 0 (0.011, 0.026), which verifies the existence of the chain mediating effect path and thus proves the tenability of Hypothesis 4. The mediating effects of implementation intention and self-identity in exercise intention and exercise behavior are shown in Figure 2.

**TABLE 5:** Analysis of mediating effects between implementation intention and self-identity.

Item	Effect	Boot SE	Boot LLCI	Boot ULCI
Exercise Intention ⇒ Implementation Intention ⇒ Exercise Behavior	0.050	0.007	0.038	0.067
Exercise Intention ⇒ Self-identity ⇒ Exercise Behavior	0.044	0.008	0.028	0.060
Exercise Intention ⇒Implementation Intention ⇒Self-identity ⇒ Exercise Behavior	0.017	0.004	0.011	0.026

*Boot LLCI refers to the lower limit of 95% interval of bootstrap sampling, and Boot LLCI refers to the upper limit of 95% interval of bootstrap sampling.*

## Discussion

### Relationship Between Exercise Intention and Exercise Behavior

The results of this study show that there is a significant positive correlation between exercise intention and exercise behavior, which is consistent with the previous relevant research results (Huang and Wu, 2021), which verifies Hypothesis 1. Downs et al. (2006) investigated 676 adolescents and found that the main motivation of individuals to participate in physical exercise is internal, and the generation of exercise intention is conducive to the realization of exercise behavior. The relationship between exercise intention and exercise behavior was explored. The results showed that the stronger the individuals’ exercise intention, the greater the possibility of their participation in exercise. It can be seen that intention is the strongest direct predictor of behavior, and exercise behavior is an important part of a healthy lifestyle. In addition, junior middle school students are in a period of rapid physical and psychological development, which is also a critical period for the formation of a healthy lifestyle (Ran and Fu, 2013). Improving their intention to physical exercise is the main intervention to promote exercise behavior. The World Health Organization (WHO) suggests that teenagers should take more than 60 min of physical exercise every day (World Health Organization, 2020). Active exercise behavior can help junior middle school students develop good exercise habits, reduce the obesity rate and myopia rate, promote the healthy development of junior middle school students’ physique, exercise all their lives, and benefit all their lives (Yao and Liu, 2021). Therefore, an in-depth exploration of the influencing factors and mechanism of junior school students’ physical exercise behavior forms the basis for advocating participation in physical exercise by teenagers, which bears vital practical and theoretical significance.

### Independent Mediating Effect of Implementation Intention and Self-Identity

This study found that implementation intention played a mediating role between exercise intention and exercise behavior, which verified Hypothesis 2. On the basis of verifying the positive prediction of exercise intention on junior school students’ exercise behavior, this study included three variables simultaneously, revealing that exercise intention is an important factor to improve implementation intention and exercise behavior. When exploring exercise intention to predict junior high school students’ exercise behavior, it is found that exercise intention has a significant positive predictive effect on implementation intention (Wang and Wang, 2021). The study also showed that implementation intention can promote exercise behavior. Wang et al. (2019) explored the impact of implementation intention on exercise behavior under different task difficulty conditions through comparative experiments. Their research results support the hypothesis that implementation intention helps improve students’ exercise behavior. More importantly, this study indicated that implementation intention plays a partial intermediary role between exercise intention and junior high school students’ exercise behavior, which means that exercise intention can directly affect junior high school students’ exercise behavior on the one hand, and indirectly affect junior high school students’ exercise behavior through implementation intention on the other hand. Some scholars believe that in addition to the conscious decision-making process, the spontaneous cognitive process without a specific purpose or consciousness may also explain behavior (such as implementation intention). The inclusion of these variables may enhance the explanatory power of consciousness on behavior (Wang and Zheng, 2020). This manifests that a strong individual intention to participate in physical exercise may contribute to a higher implementation intention. Ajzen (2011), the proponent of the planned behavior theory, also believes that on the basis of intervention based on the planned behavior theory, increasing intervention on implementation intention can effectively narrow the gap between goals and actions. Higher implementation intention can facilitate the satisfactory completion of the exercise plan well, while lower implementation intention will affect the implementation of the exercise plan. Therefore, as far as the relationship between exercise intention and exercise behavior of junior school students is concerned, the role of implementation intention should be given great importance.

This study has also found that self-identity has a partial mediating effect between exercise intention and exercise behavior, which has verified Hypothesis 3. This is consistent with the research results that exercise intention promotes self-identity (Dong, 2013), and self-identity has a positive predictive effect on exercise behavior (Jackson et al., 2003; de Bruijn and van den Putte, 2012). In the meantime, the study has examined the relationship between them, and related results show that exercise intention is not only a crucial factor to enhance self-identity but also a significant element to promote exercise behavior of junior school students. To transform exercise intention into behavior more effectively, improving the level of self-identity also proves to be an effective means to achieve this objective. Students with high self-identity have stronger self-regulation and control than others (Choi and Kim, 2021). When self-identity is cultivated, junior school students can “redefine themselves” with a more comprehensive and objective self-awareness and believe that they are capable of completing exercise tasks, resulting in more positive exercise behavior (Hardcastle and Taylor, 2005).

### Chain Mediating Effect of Implementation Intention and Self-Identity

This study has further found that implementation intention and self-identity have a chain mediating effect between exercise intention and exercise behavior of junior school students, which has verified Hypothesis 4. This is consistent with the existing research results that implementation intention facilitates self-identity (Fekadu and Kraft, 2001). Exercise intention can provide the condition of self-identity for junior school students, while implementation intention acts as the driving factor of self-identity. The stronger the relevance of implementation intention, the larger the possibility of individuals conducting practical action. Individuals with a high level of self-identity bear a high level of cognition of themselves, which enables them to reflect on the decisions they make and to actively complete the tasks within their capability. Therefore, higher exercise intention of junior school students contributes to higher implementation intention and self-identity level, resulting in positive exercise behavior.

Accordingly, the chain mediating effect of implementation intention → self-identity in this study is feasible, which can have a partial mediating effect on the prediction of exercise awareness on exercise behavior. Therefore, taking implementation intention and self-identity as the “third variable” to bridge the gap between exercise intention and exercise behavior is helpful to explain and predict the complex mechanism of intention behavior transformation and has certain guiding value for improving the exercise behavior of junior school students. To a certain extent, analysis results of chain mediation effect in this study reminds physical educators that in the process of advocating junior school students to transform from “intention” to “implementation,” thereby promoting exercise behavior and enhancing exercise intention in junior school students, while lifting implementation intention and self-identity is still the most significant path.

## Conclusion

This study explores the relationship among exercise intention, implementation intention, self-identity, and exercise behavior. Through the in-depth analysis of the questionnaire data of junior middle school students, it can be seen that exercise intention, implementation intention, self-identity, and exercise behavior have a significant positive correlation; exercise intention, implementation intention, and self-identity have a significant positive correlation; and exercise intention and implementation intention have a significant positive correlation. The results of this study show that behavioral intention and self-identity have a significant mediating effect between exercise intention and exercise behavior. It contains three mediating paths: the independent mediating effect of execution intention, the independent mediating effect of self-identity, and the chain mediating effect of implementation intention and self-identity. Among them, the proportion of the self-identity effect is lower than that of implementation intention. This study reveals that junior middle school students’ exercise behavior is affected by their own level of implementation intention and self-identity to a certain extent. Therefore, in the process of improving junior middle school students’ exercise behavior, it is recommended to take promoting the physical and mental health development of junior middle school students as a foothold, starting from strengthening junior middle school students’ awareness of physical exercise, paying attention to the level of junior middle school students’ implementation intention and self-identity, and promoting the improvement of junior middle school students’ exercise behavior by improving the level of junior middle school students’ implementation intention and self-identity, so as to enable junior middle school students to develop lifelong sports awareness and good exercise habits.

### Limitations and Future Directions

This study explores the relationship between exercise intention and exercise behavior of junior school students, constructs a chain mediating model, and reveals the internal mechanism of the impact of exercise intention on exercise behavior of junior school students, which has certain guiding values for bridging the gap between exercise intention and exercise behavior and has also provided a preliminary basis for studying the causal relationship between variables. However, this study adopts the retrospective and subjective reporting methods to evaluate the exercise behavior of junior school students, which is vulnerable to individual subjectivity, and it is still unable to infer the causal relationship between variables. In the future, longitudinal tracking or experimental intervention design can be applied to explain the impact of exercise intention on junior school students’ exercise behavior more effectively. In addition, this study only inspects the mediating effects of implementation intention and self-identity on exercise intention and exercise behavior of junior school students, neglecting other possible mediating variables in reality, such as emotion, self-efficacy, and exercise social support, which need to be further studied. The follow-up research should highlight the concept of “strengthening physique and promoting health” and deeply explore the “dose effect” of the physical exercise program and the influence of relevant variables, so as to assist junior school students to obtain more supporting paths.

## Data Availability Statement

The original contributions presented in this study are included in the article/Supplementary Material, further inquiries can be directed to the corresponding authors.

## Ethics Statement

The studies involving human participants were reviewed and approved by the Human Research Ethics Committee of Huaibei Normal University. Written informed consent to participate in this study was provided by the participants’ legal guardian/next of kin.

## Author Contributions

YC and S-JY designed the study, collected and analyzed the data, and wrote the manuscript. Q-SM, K-LG, WS, and CL revised the manuscript. All authors contributed to the article and approved the submitted version.

## Conflict of Interest

The authors declare that the research was conducted in the absence of any commercial or financial relationships that could be construed as a potential conflict of interest.

## Publisher’s Note

All claims expressed in this article are solely those of the authors and do not necessarily represent those of their affiliated organizations, or those of the publisher, the editors and the reviewers. Any product that may be evaluated in this article, or claim that may be made by its manufacturer, is not guaranteed or endorsed by the publisher.

## Appendix

### Questionnaire items

**Table:**

Exercise intention	
In the next 2 weeks, exercise for at least 20 min at least three times a week.	
…I plan to do the above physical exercises	()
…I intend to do the above physical exercises	()
…I hope to do the above physical exercises	()
**Implementation intention**	
I have planned the time to participate in physical exercise next week	()
I have planned the place to participate in physical exercise next week	()
I have planned the way to participate in physical exercise next week	()
I have completed physical exercise within the planned time	()
I have completed physical exercise at the planned location	()
I have finished my physical exercise as planned	()
**Self-identity**	
If I give up exercise, I will feel very defeated.	()
I like physical exercise.	()
Taking part in physical exercise is in line with my character.	()
I think I am healthy and do not need exercise	()
It is important for me to take part in physical exercise.	()
I tend to take part in physical exercise.	()
I consider myself a good athlete.	()
Taking part in physical exercise is an important part of my life.	()
People around me think I am a person who likes physical exercise.	()
In terms of personality, I am the kind of person who must take part in physical exercise.	()
I think I’m in good shape and don’t need to take part in physical exercise.	()
Taking part in physical exercise is a way of life for me.	()
**Exercise Behavior**	
**What is the intensity of your physical exercise?**	
□Slight movement □A low-intensity, less intense exercise □A more intense, sustained exercise of moderate intensity □Intense but not sustained exercise with rapid breathing and excessive sweating □A high-intensity, long-lasting exercise with rapid breathing and excessive sweating	
**How many minutes do you spend on the above intensive sports activities?**	
□Less than 10 min □11–20 min □21–30 min □31–59 min □More than 59 min	
**How many times do you do the above sports activities in a month?**	
□Less than once a month □Two to three times a month □Once or twice a week □Three to five times a week □About once a day	
